# Enhanced terahertz fingerprint detection with ultrahigh sensitivity using the cavity defect modes

**DOI:** 10.1038/s41598-017-13612-9

**Published:** 2017-10-13

**Authors:** Xiaomei Shi, Zhanghua Han

**Affiliations:** Lab of Terahertz Photonics, China Jiliang University, Hangzhou, 310018 China

## Abstract

We report a new scheme of realizing terahertz fingerprint detection with ultrahigh sensitivity. Instead of using the direct absorption of terahertz through a bare sample in the regular transmission scheme, a cavity mode resonating at the characteristic frequency of the sample is used and due to the high dependence of the cavity mode transmission on the material loss, an amplified transmission decaying is observed when the sample is loaded into the cavity. Furthermore, this scheme retains the feature of substance identification. A one-dimensional photonic crystal cavity is used as the example for the detection of α-lactose and an efficient detection of 10 nm α-lactose can be achieved, which corresponds to 1/57000 of the free space wavelength at the characteristic frequency of 0.529 THz, exhibiting a sensitivity over 400 times higher than the regular transmission method.

## Introduction

Terahertz (abbreviated as THz and typically defined between 0.1 THz and 10 THz) radiations have many advantages over the electromagnetic waves in other frequencies due to their favorable properties like transparency in most dielectrics, low photon energy and nonionizing features, which may promise many potential applications ranging from fundamental sciences to practical applications^1–3^. Especially, many chemicals and molecules have their characteristic absorption frequencies located in the terahertz regime resulting from their rotation, intra- and inter-molecular vibrations, suggesting that terahertz technology can work as a unique tool for chemical identification^4–6^. This technique, well-known as fingerprint detection, lays the foundation for many applications of terahertz in security checking (like drugs and explosives), biomedical diagnosis and pharmaceutic industry. However, due to the high contrast between the nanometer-scale size of most molecules and terahertz wavelengths (from tens to a few hundreds of microns), the interactions between molecules and THz radiations are extremely weak and a large volume of chemicals are usually required to have an observable absorption for identification. For example, in the mostly-used transmission scheme for THz fingerprint detection, the sample is normally made into powder and then compressed into pellets with a thickness of several millimeters^7^. There are many circumstances in which the sample is not abundant or it is required to use as few sample as possible, e.g. in medical diagnosis. Thus, a substantial improvement of the sensitivity in the THz fingerprint detection is still necessary to further broaden this technique in practical applications. Many approaches have been attempted in this respect, for example to use resonating antennas, metamaterials or InSb-based plasmonic gratings^8–11^ in the transmission mode to enhance the local electric field so that the absorption cross section of the chemicals can be amplified. Other examples include the exploration of new working principles like hybridization induced transparency^12^ or using waveguiding structures^13–15^ to increase the interaction length between molecules and terahertz radiations for a fixed amount of sample. However, with all these reported approaches, to get an observable dependence of the transmission at the characteristic frequencies on the sample amount, the required sample thickness is on the order of several micrometers^10,11^.

## Working principal and results

**Figure 1:**
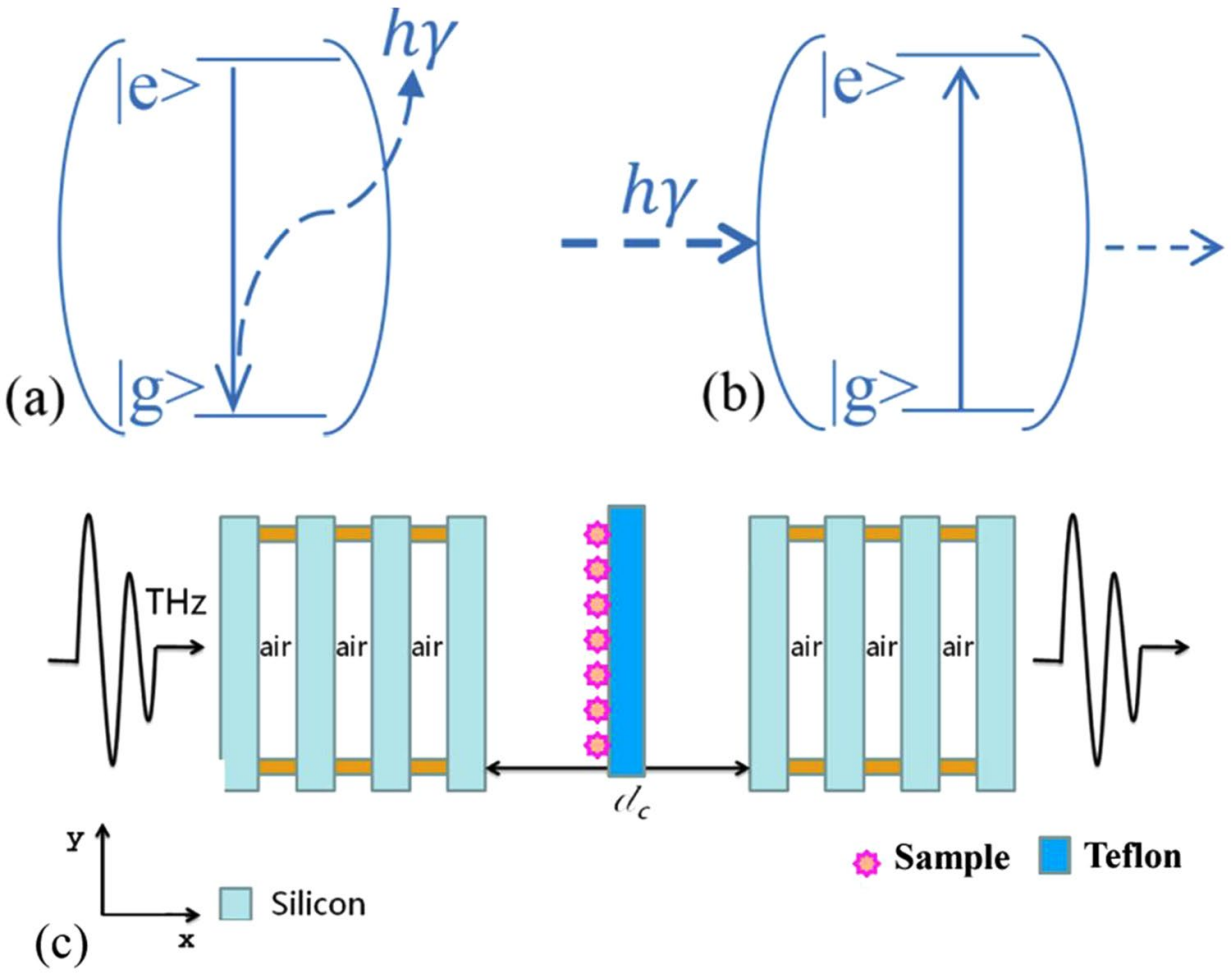
Schematics for (**a**) the change of spontaneous rate of an emitter placed inside a cavity in the weak coupling regime; (**b**) the amplified transmission drop of a cavity mode with the frequency matching the characteristic frequency of the sample loaded into the cavity and (**c**) the 1D PC cavity composed of one central defect layer between two distributed Bragg reflectors made from four silicon stacks. The yellow parts are double-sided adhesive used to connect different parts while the area located in the center of the defect layer indicates the loading of sample on a Teflon film.

In this paper, we propose a novel idea of realizing THz fingerprint detection with an ultrahigh sensitivity and the sample as thin as a few tens of nanometers can be easily and steadily captured. Basically, to realize the functionality of fingerprint detection in the regular transmission scheme using a THz spectroscopy, one needs the information for two features. One is the characteristic frequencies which are the spectral signatures of a certain sample, manifesting them as resonances in the transmission spectrum; the other is the change of the transmittance at these frequencies (usually normalized using the transmittance through the sample to that through vacuum) which implies the amount of samples included in the measurement. It is well-known in the weak-coupling regime that when an emitter is placed into a man-made cavity structure which has a designed resonance frequency corresponding to the energy difference between the ground and excited states of the emitter, as shown in Fig. 1(a), the spontaneous emission rate of the emitter can be enhanced due to Purcell effect^16^. Inspired by this picture, we conceived an idea of using a similar structure but with the reverse process for THz fingerprint detection with ultrahigh sensitivity. The detection scheme is schematically shown in Fig. 1(b). A cavity structure is designed to have a resonating frequency *f*_0_ which matches one of the characteristic frequencies of the target sample. When the sample is absent, the transmission through the cavity structure will exhibit a peak at *f*_0_. If a thin target sample is present, due to the intrinsic absorption of the sample at *f*_0_, the transmission will experience a drop and the resonance frequency will remain roughly at *f*_0_ due to the little optical path length change from the thin sample. This drop, however, will be much higher than the transmission through a bare sample due to the cavity effect. When another sample with an absorption frequency away from *f*_0_ is considered, it works as a dielectric with small or negligible loss around *f*_0_. So, when it is loaded into the cavity, the resonance will be spectrally shifted but its transmission won’t be significantly affected. One can see that using this scheme the sample can be identified while its amount can be measured with high sensitivity as well. The high dependence of the cavity mode transmission on the sample amount makes it possible to realize the quantitative detection easily.

One can also interpretate the working principal using another way. One knows that the power transmission through a sample can be calculated using the following equation:

1 $${\rm{T}}={e}^{-4\pi k(\omega )L/\lambda }$$

where *k(ω*) is the imaginary part of the sample refractive index, which reaches its maximum at the sample characteristic frequencies, *L* is the sample thickness and λ is the free space wavelengths. Due to large values of λ in the terahertz band, *L* must be large to achieve an observable decrease in *T* at resonance. However, when the sample is located in a cavity resonating at the sample characteristic frequency, the THz radiation at resonance will bounce back and force for multiple times, interacting with the sample and resulting in an effective sample thickness *L*_*eff*_ much larger than the original *L*.

**Figure 2:**
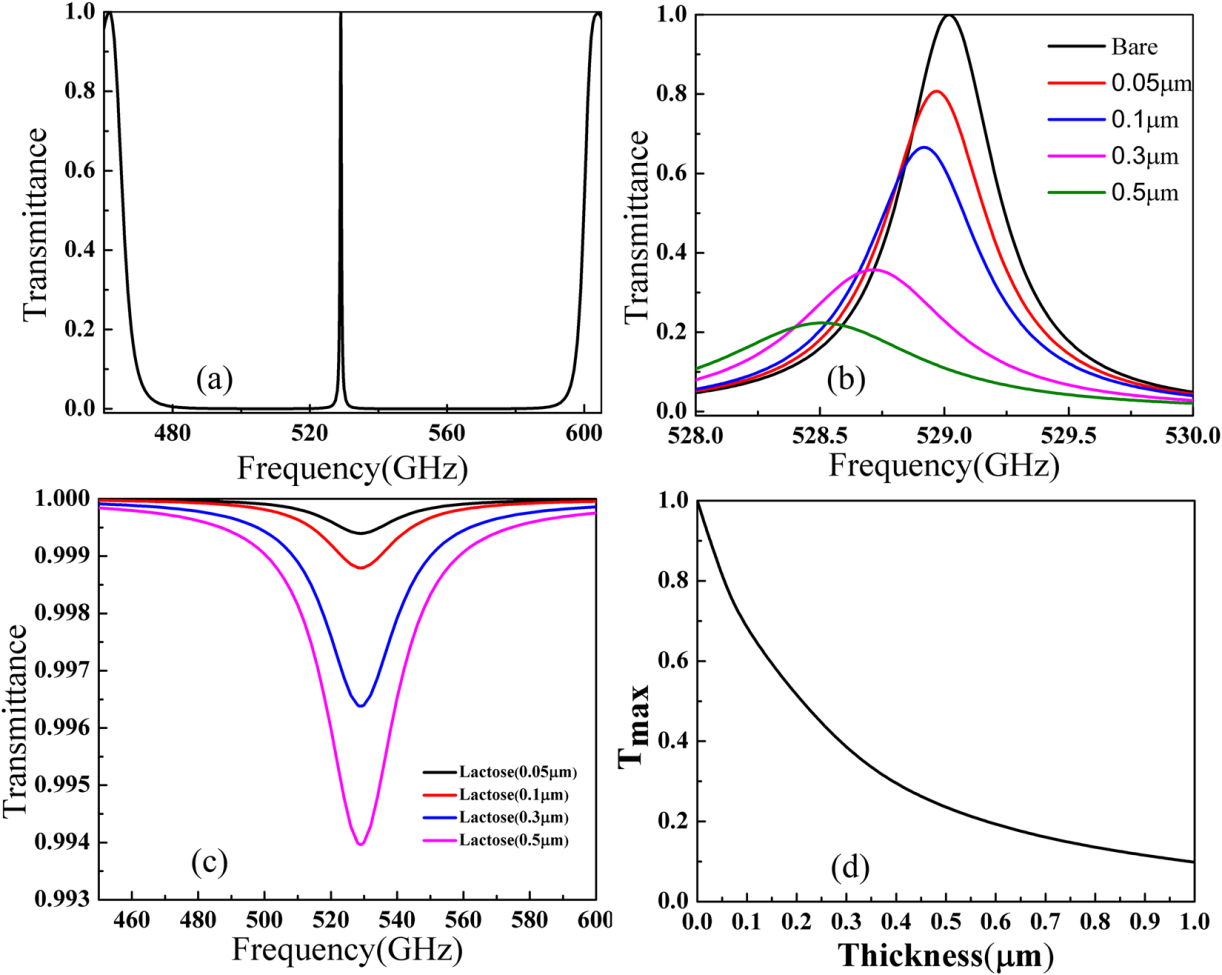
(**a**) Simulated transmission spectrum of the bare photonic crystal cavity with *d*_*c*_ = 268 μm; *t* = 100 μm; *w* = 233 μm. (**b**) Transmission of 1D photonic crystal cavity with different thickness of α-lactose   loaded onto the left side of the Teflon film embedded in center of this cavity. (**c**) The transmission through a bare α-lactose  layer with different thicknesses. (**d**) Dependence of the maximum value of transmittance through this cavity at resonance on the thickness of α-lactose.

In the following part, we demonstrate the capability of the proposed scheme for THz fingerprint detection using a one-dimensional (1D) photonic crystal (PC) cavity structures as an example. Composed of Si stacks, these structures have been experimentally investigated by us and the measured transmission spectrums demonstrate cavity resonances with high quality factors^17^. As shown schematically in Fig. 1(c), the structure consists of one central defect cavity between two parallel Bragg reflectors distributed on both sides. Each Bragg mirror is composed of four silicon stacks separated by air. Defect mode in the photonic bandgap can be formed due to the Fabry-Perot reflections inside the cavity. One thin Teflon film with the thickness of 50 µm is placed in the center of the cavity to work as the accommodation place for the sample. The refractive index of silicon, Teflon and the air layers are 3.44, 1.46 and 1, respectively. The length of the defect cavity (*d*_c_) determines the numbers and frequencies of defect modes in this 1D photonic crystal cavity^17^. For simplicity, we selected a length of this defect cavity (*d*_*c*_) being 268 μm, which combing with other parameter, results in a transmission peak around 0.529 THz. This frequency is a signature of α-lactose, which will be used as the sample representative for the fingerprint detection in this paper. The thickness of the silicon layers is *t* = 100 μm and air layer thickness between them *w* = 233 μm. The transmission characteristics of this photonic crystal cavity are numerically simulated with the finite element method (FEM). In the calculations, the incident THz plane wave is set be normal to the structure with electric field along the *y* direction. The calculated transmission spectrum of the bare photonic crystal cavity is shown in Fig. 2(a) which clearly illustrates the existence of the cavity mode at 0.529 THz. An enlarged view of the transmission peak can be seen as the black line in Fig. 2(b), which shows that the peak is very sharp with a full-width at half-maximum bandwidth of 0.45 GHz resulting in a quality factor more than 1150 and the transmittance at resonance through this structure can approach 100%.

When a thin layer of α-lactose is loaded on one side of Teflon film into this cavity as shown in Fig. 1(c), the peak transmission will be significantly affected by the intrinsic loss of it. The permittivity of α-lactose is modelled using with a series of Lorentzian oscillators as follows^18^:

2 $${{\boldsymbol{\varepsilon }}}_{{\boldsymbol{r}}}={{\boldsymbol{\varepsilon }}}_{\infty }+\sum _{{\boldsymbol{p}}=1}^{\infty }\frac{{\rm{\Delta }}{{\boldsymbol{\varepsilon }}}_{{\boldsymbol{p}}}{{\boldsymbol{\omega }}}_{{\boldsymbol{p}}}^{2}}{{{\boldsymbol{\omega }}}_{{\boldsymbol{p}}}^{2}-{{\boldsymbol{\omega }}}^{2}-{\boldsymbol{j}}{{\boldsymbol{\gamma }}}_{{\boldsymbol{p}}}{\boldsymbol{\omega }}}$$

where ${{\rm{\varepsilon }}}_{\infty }\,$denotes the off-resonance background permittivity of α-lactose, ${\omega }_{p}\,$and ${{\rm{\gamma }}}_{{\rm{p}}}\,\,$are the angular frequency and damping rate of each absorption oscillation respectively and ${{\rm{\varepsilon }}}_{{\rm{p}}}$ is the oscillation strength factor. For simplicity only the first order absorption resonance of α-lactose  at 0.529 THz is considered and the other parameters are as follows ${{\rm{\varepsilon }}}_{\infty }=3.145$, ${{\rm{\gamma }}}_{p}$= 1.59×10^11^ *rad* s^−1^ and ${{\rm{\varepsilon }}}_{p}=0.052$, which together gives a calculated permittivity close to the empirical values^19^. When the α-lactose layer as thin as 0.05 μm is loaded on the left side of the Teflon film, one can see from the red line in Fig. 2(b) that the transmittance at resonance drops by 20% dramatically from around 100% without lactose to be 80.7% while the position of the peak remains almost unchanged. This is due to a significant dependence of the cavity mode on the material loss inside the cavity at resonance and a negligible change of the overall optical path due to the small thickness of  α-lactose. As can be seen in Fig. 2(b), a successive increase of the α-lactose thickness will result in a further decrease of the transmittance at resonance, followed by a weak resonance shift due to a larger change in the optical path. The overall transmittance at resonance as a function of the α-lactose thickness is shown in Fig. 2(d) which exhibits an exponential decaying behavior. One may also notice that the resonance positions in Fig. 2(b) for different α-lactose thicknesses follow the trend of the black line which is the original transmission resonance of the bare PC cavity. This implies, to get a higher sensitivity of the transmittance drop as a function of α-lactose thickness, one should design an original cavity resonance with a higher quality factor. Actually, with the current cavity structure, if one assumes 5% is the limit of transmittance drop that can be steadily observed, our calculations show that value corresponds to a α-lactose thickness as small as 10 nm, which characterizes the ultimate resolution of our approach. One can also see in Fig. 2(d) that the resonance transmittance drop saturates at a α-lactose thickness around 1 μm. This dynamic range is determined by the thickness when the resonance peak shifts beyond the original cavity resonance in Fig. 2(b). In this aspect, to get a higher dynamic range, one should use a PC cavity resonance with a lower quality factor. A trade-off between the sensitivity and the dynamic range should always be balanced and this can be adjusted by changing the quality factor of the cavity mode.

For comparison, we also plot in Fig. 2(c) the transmission through such thin-layers of bare α-lactose. It is obvious that at resonance the drop in transmittance for a α-lactose thickness of 0.05 μm is only 0.06% and the number only increases to 0.34% and 0.59% even if the α-lactose thickness is 0.3 μm and 0.5 μm respectively. If one compares the numbers, it is found that the addition of the 0.05 μm α-lactose in the 1D photonic crystal cavity will introduce a drop in transmittance at the characteristic frequency which is about 321 times of that in a bare α-lactose layer of the same thickness. This number increases to roughly 415 at the α-lactose thickness of 10 nm. In other words, the transmittance drop of α-lactose can be amplified by 415 times using our scheme. More importantly and worth noting, these transmittance drops indicated in Fig. 2(c) are probably not accessible with most terahertz spectroscopies due to the signal-to-noise issue while the drops in Fig. 2(b) can be steadily measured.

**Figure 3:**
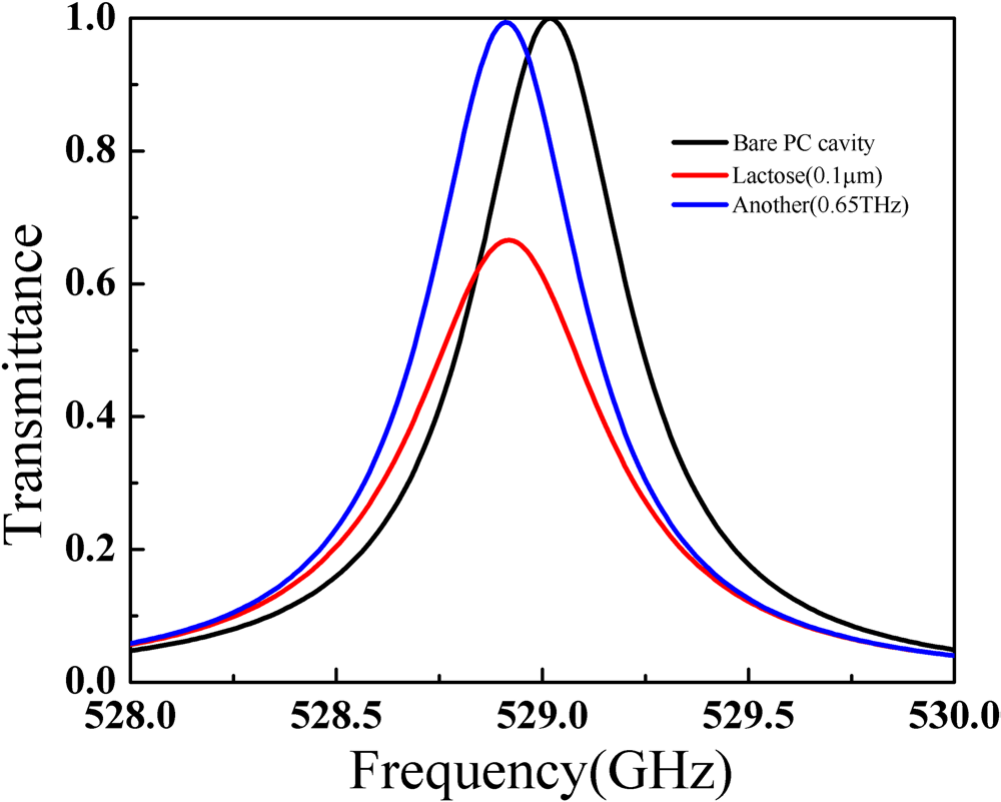
A comparison of the transmission spectrum for three different cases: the bare PC cavity, the PC cavity with 0.1 μm α-lactose and the PC cavity with 0.1 μm of another sample with the absorption frequency at 0.65 THz.

To further demonstrate that our scheme keeps the feature of substance identification similar to the conventional transmission scheme, we replace the α-lactose with another sample which has a different intrinsic absorption frequency. A new value of 2π*0.65 THz which is beyond the bandgap of the cavity is used for ${\omega }_{{{\rm{p}}}_{}}\,$in equation (2) while all the other parameters are assumed unchanged. The thickness of the new sample is 0.1 μm and the results of transmission spectra for three structures, the bare PC cavity, the PC cavity with 0.1 μm of α-lactose, and the PC cavity with 0.1 μm of the new sample with absorption at 0.65 THz, are shown in Fig. 3 for comparison. It can be seen that the transmittance for the PC cavity designed to identify the existence of α-lactose, only decreases when 0.1 μm of α-lactose is loaded while for the new sample the resonance of the PC cavity only experiences a red shift of 0.1 GHz but with no evident decrease in the transmittance. This is because the new sample with an absorption frequency away from that of α-lactose will only introduce an additional optical path around 0.529 THz in the cavity.

## Discussions

**Figure 4:**
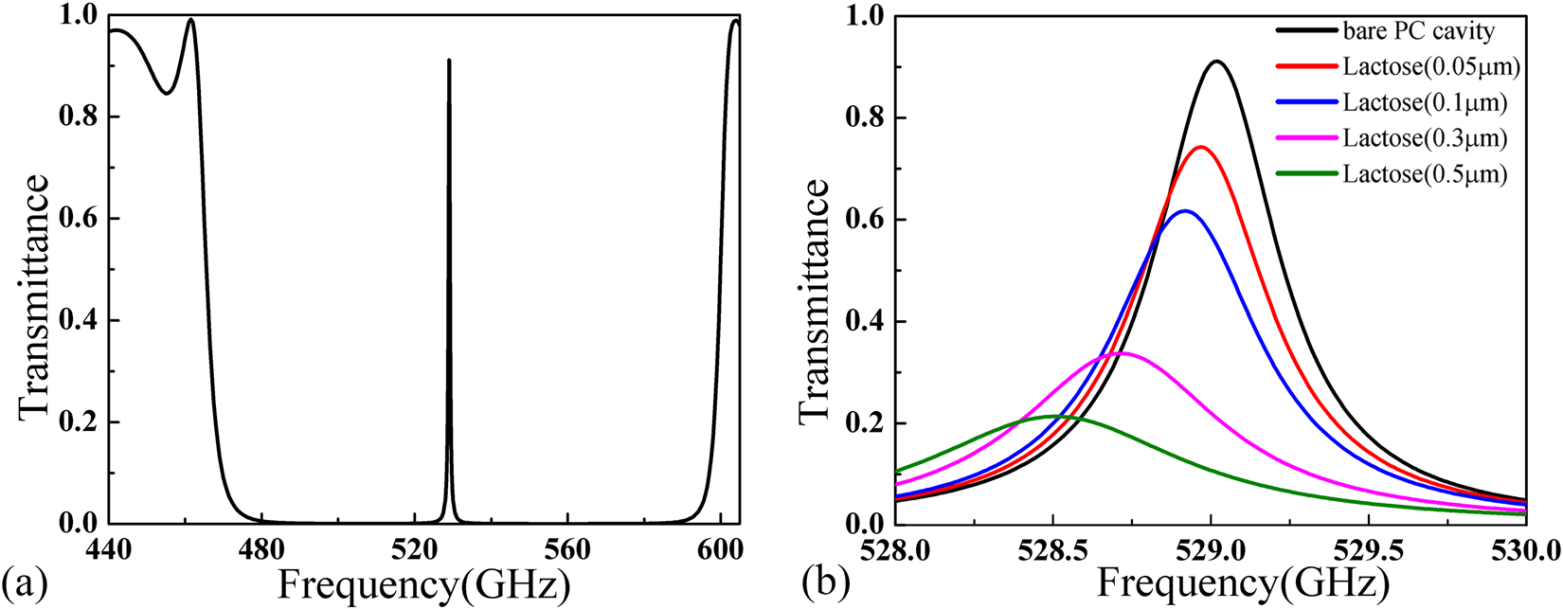
The transmission spectrum for (**a**) the bare PC cavity embedded a Teflon film with lossy silicon stacks and (**b**) loaded with different thickness of α-lactose. The imaginary part of silicon permittivity is assumed to be 0.001j.

In the above calculations, the silicon is assumed to be lossless. For practical applications, even the high-resistivity silicon may exhibit some losses in the terahertz frequencies. Since the material loss is critical in determining the property of a photonic cavity, we further consider the case when the material loss of silicon is taken into account. The calculated transmission spectrum of this bare PC cavity when the imaginary part of silicon permittivity is assumed being 0.001j is shown in Fig. 4(a). One can see that the transmittance at resonance is about 91% which is slightly lower than the previous value presented in Fig. 2(a). The transmission spectra when different thicknesses of α-lactose loaded into this lossy cavity are shown in Fig. 4(b). Although the cavity performance is slightly deteriorated by the material loss, one can see that the scheme of enhanced detection using the cavity still works. When the thickness of α-lactose is only 0.05 μm, the transmittance drops by 16.7% (from 91% to 74.3%), exhibiting sensitivity more than 270 times higher than regular transmission method. With an even higher material loss, the transmittance may experience further decrease; however similar dependence of peak transmittance on the thickness of loaded sample will be reproduced.

**Figure 5:**
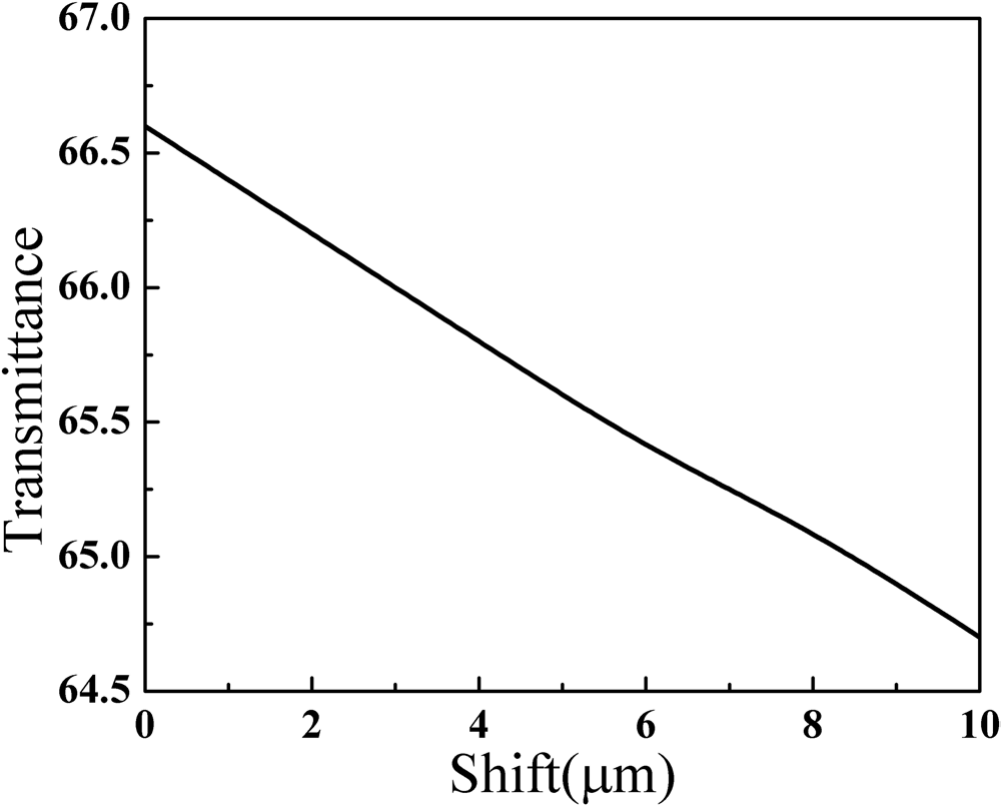
The peak transmittance as a function of the Teflon film shift from the cavity center.

Due to the formation of the standing waves in the cavity, the lateral positions of the loaded α-lactose on Teflon film will also affect the sensitivity. The highest sensitivity will be expected when the sample is loaded at the maximum electric field of the cavity, which is the physical center of the cavity for our investigated defect mode. Figure 5 presents the peak transmittance as a function of the shift between the centers of the Teflon film and the cavity, when the loaded α-lactose thickness is 0.1 μm. One can clearly see that the peak transmittance decreases by less than 2% when the Teflon film is within 10 μm from cavity center. Since the position can be easily adjusted with a resolution of several microns using some commercial displacement systems, one can conclude that the location of the quartz crystal is not so critical in this setup.

**Figure 6:**
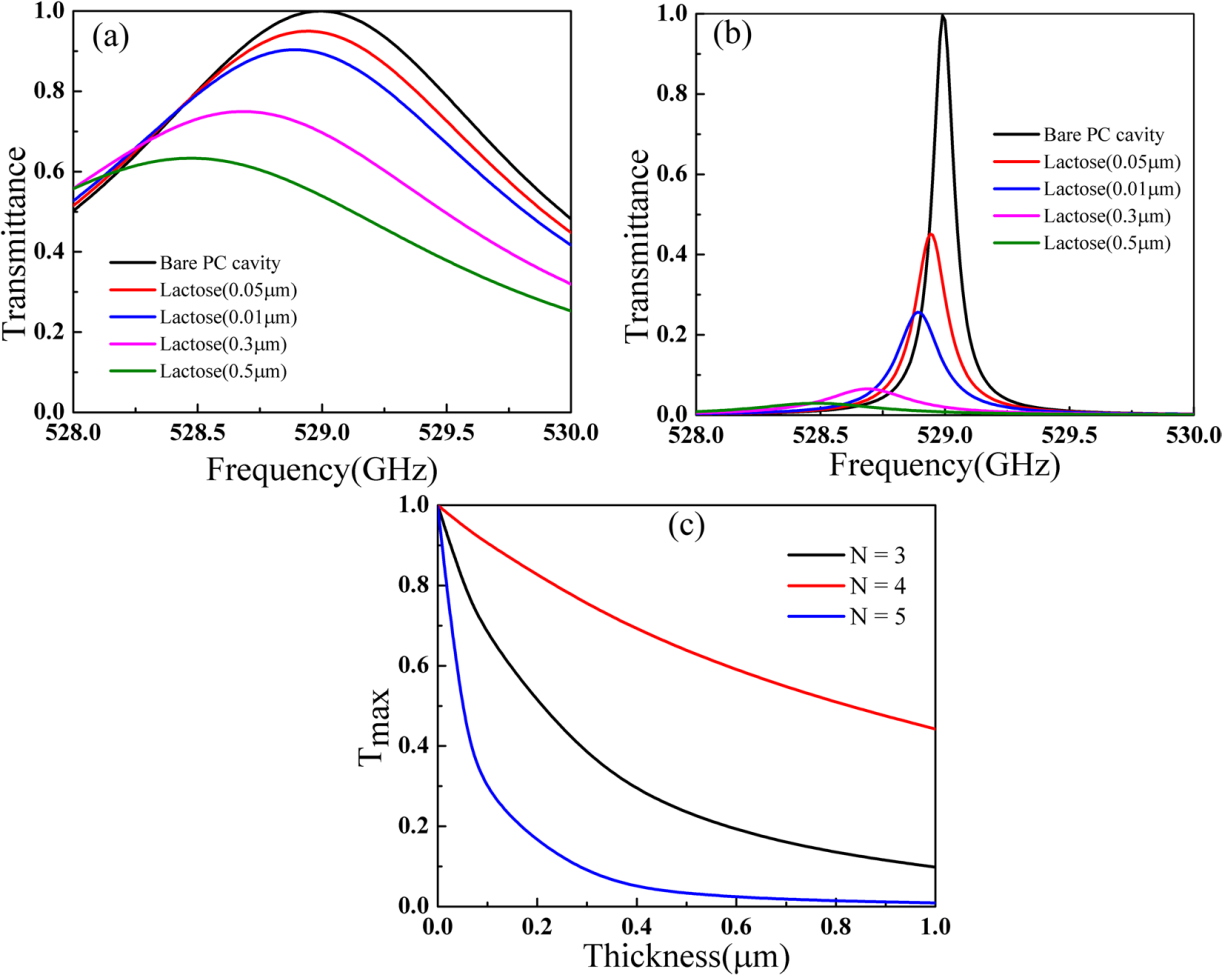
Transmission of 1D photonic crystal cavity with different thickness of α-lactose through cavities composed of different numbers of Si stacks, N = 3 for (**a**) and N = 5 for (**b**); (**c**) the peak transmittance as a function α-lactose.

From the results in Fig. 2(b), one can see that the dependence of peak transmittance on the α-lactose thickness is very sensitive to the quality factor of the defect mode. Thus, one can achieve sensors with different performances by adjusting the quality factor of the defect mode. To demonstrate this feasibility, we used the same PC cavity as shown in Fig. 1(c) but changed the number of silicon stacks on either side of the cavity from *N* = 4 to *N* = 3 and 5 respectively. The quality factors for the two cases are found to be 270 and 5076 respectively. A larger number of Si stacks will result in the defect mode with a higher quality factor because the leaking of THz radiation through the PC structure is weaker. Figure 6(a),(b) present the transmission spectra around 0.529 THz for the two cavities with different quality factors. The trend lines of peak transmittance at resonance for the cavities with *N* = 3, 4, 5 are plotted in Fig. 6(c). One can see that with a higher quality factor, the peak transmittance drops fasters as a function of sample thickness, suggesting a higher sensitivity. However, the peak transmittance also saturates at a smaller sample thickness, which implies that a higher quality factor is associated with a smaller dynamic range of sensing.

**Figure 7:**
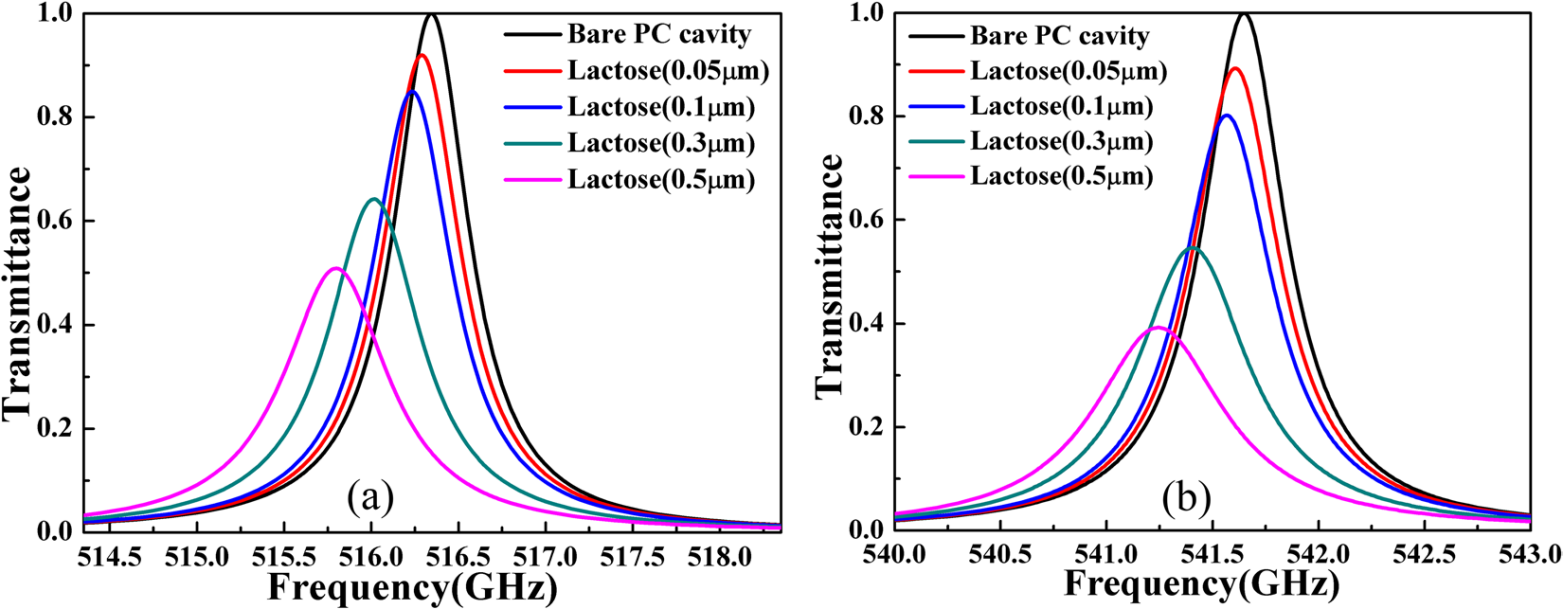
Transmission spectra for different thickness of α-lactose when the cavity defect mode matches frequencies at two ends 0.529THz-0.5* 25.3 GHz in (**a**) and 0.529 THz + 0.5* 25.3 GHz in (**b**) of the absorption bandwidth.

As a final remark, we know that the all the sample absorption resonances have certain bandwidths around the central frequencies. For example, the bandwidth for the α-lactose at the resonance of 0.529 THz is 25.3 GHz. For all the above calculations, we assume that the cavity defect mode has a resonance frequency matching 0.529 THz exactly. However, even when there is some mismatch between the cavity defect mode and the characteristic frequency, the proposed scheme still works. Figure 7 presents the transmission spectra for two cases when the defect mode frequency matches the two frequencies at both ends of the bandwidth, i.e. 0.529 THz ± 0.5* 25.3 GHz. One can see that the enhanced sensitivity still works even when the designed defect mode doesn’t match the sample characteristic frequency, showing the robustness of the design.

## Conclusion

Although a simple structure of 1D PC cavity is used in this paper to demonstrate the working principle of our proposed idea to realize terahertz fingerprint detection with unpresented sensitivity, we need to emphasize that the idea can be extended easily to other cavity structures working in the terahertz frequencies, like Si based waveguide ring resonators or photonic crystal cavities. Some on-chip cavity structures can even add more assets because the interaction length between the cavity mode and the sample is further increased.

In a summary, we have presented in this paper a new scheme to realize the THz fingerprint detection with ultrahigh sensitivity. Using a resonance mode matching the absorption of α-lactose in a 1D PC cavity to amplify the influence of the α-lactose intrinsic absorption to the change of transmittance at the resonance peak, a transmittance drop of more than a few hundred times can be obtained compared to that through a bare α-lactose layer. With this method, an efficient detection of a thin α-lactose layer with a thickness as small as 10 nm can be easily and steadily achieved. This thickness corresponds to only 1/57000 of the free space wavelength at the characteristic frequency of 0.529 THz. A steady identification of such a thin layer of sample makes it possible to realize the recognition of a monolayer of molecules, suggesting huge potential for terahertz application in medical diagnosis. The proposed design can be easily extended to other substances by simply adjusting the cavity parameters to match the absorption frequencies of new materials. This versatility, combined with the ultra-high sensitivity, will make a significant impact on the current terahertz technology. It lays out the foundation for terahertz sensing in circumstances where high sensitivity is essential while still retaining the unique property of characteristic frequency identification. We believe that this technique really broadens the application range for the terahertz technology and paves the way for the further application of terahertz spectroscopy in biomedical area.
